# JMJD3: a critical epigenetic regulator in stem cell fate

**DOI:** 10.1186/s12964-021-00753-8

**Published:** 2021-07-03

**Authors:** Yuanjie Ding, Yuanchun Yao, Xingmu Gong, Qi Zhuo, Jinhua Chen, Miao Tian, Maryam Farzaneh

**Affiliations:** 1 School of Medicine, Jishou University, Jishou, 416000 China; 2 Key Laboratory of Hunan Forest Products and Chemical Industry Engineering, Jishou University, Zhangjiajie, 427000 China; 3 Fertility, Infertility and Perinatology Research Center, Ahvaz Jundishapur University of Medical Sciences, Ahvaz, Iran

**Keywords:** Pluripotent stem cells, JMJD3, Pluripotency, Reprogramming, Differentiation

## Abstract

The Jumonji domain-containing protein-3 (JMJD3) is a histone demethylase that regulates the trimethylation of histone H3 on lysine 27 (H3K27me3). H3K27me3 is an important epigenetic event associated with transcriptional silencing. JMJD3 has been studied extensively in immune diseases, cancer, and tumor development. There is a comprehensive epigenetic transformation during the transition of embryonic stem cells (ESCs) into specialized cells or the reprogramming of somatic cells to induced pluripotent stem cells (iPSCs). Recent studies have illustrated that JMJD3 plays a major role in cell fate determination of pluripotent and multipotent stem cells (MSCs). JMJD3 has been found to enhance self-renewal ability and reduce the differentiation capacity of ESCs and MSCs. In this review, we will focus on the recent advances of JMJD3 function in stem cell fate.

**Video Abstract**

**Supplementary Information:**

The online version contains supplementary material available at 10.1186/s12964-021-00753-8.

## Background

In the human genome, the sequence of DNA and epigenetic mechanisms such as DNA methylation, histone modification (methylation, phosphorylation, acetylation, adenylation, ADP ribosylation, and ubiquitination), and chromatin remodeling are thought to alter gene expression [1, 2]. It has been discovered that the aberrant expression of histone methylation leads to demethylation and abnormal histone methylation, which contributes to migration, invasion, and carcinogenesis, during cancer development [3, 4]. The trimethylation of histone H3 at lysine-27 (H3K27me3) is an epigenetic modification that contributes to epigenetic silencing and influences the development of normal and abnormal cells [5–7]. The accumulation of H3K27me3 is essential for the recruitment of transcription factors (TFs) to DNA [8, 9]. The Jumonji domain-containing protein-3 (JMJD3, also known as KDM6B) is a histone demethylase that regulates H3K27me3-mediated gene repression and removes H3K27me3 [10, 11]. JMJD3 has been studied extensively in immune diseases, cancer, and tumor development [12–16]. Recent studies have illustrated that JMJD3 plays an important role in cell fate determination of pluripotent and multipotent stem cells [11, 17, 18]. Human pluripotent stem cells (PSCs), including embryonic stem cells (ESCs) and induced pluripotent stem cells (iPSCs), have the ability to divide and give rise to all cells of the tissues of the body under specific conditions [19, 20]. Human PSCs are very useful in the field of disease modeling, drug screening, and cell-based regenerative medicine for many diseases [21–23]. Human ESCs can be derived from the inner cell mass (ICM) of the human blastocyst [24]. Human iPSCs are generated by introducing exogenous factors (OCT4, SOX2, KLF4, and c-MYC) into somatic cells [25]. Mesenchymal stem cells (MSCs) are multipotent stem cells with the capacity to differentiate into various cell types such as adipocytes, osteocytes, neurocytes, hepatocytes, and chondrocytes [26, 27]. MSCs can be obtained from bone marrow (BM), dental pulp (DP), adipose tissues (AT), and umbilical cord blood (UCB) [28, 29]. Several recent studies have shown that JMJD3 plays an important role during the transition of ESCs and MSCs into specialized cells or the reprogramming of somatic cells to iPSCs [11, 30]. JMJD3 has been found to enhance self-renewal ability and reduce the differentiation capacity of pluripotent and multipotent stem cells [12, 31, 32]. In this review, we will focus on the recent advances of JMJD3 function in stem cell fate.

## Structure and function of JMJD3

**Fig. 1:**
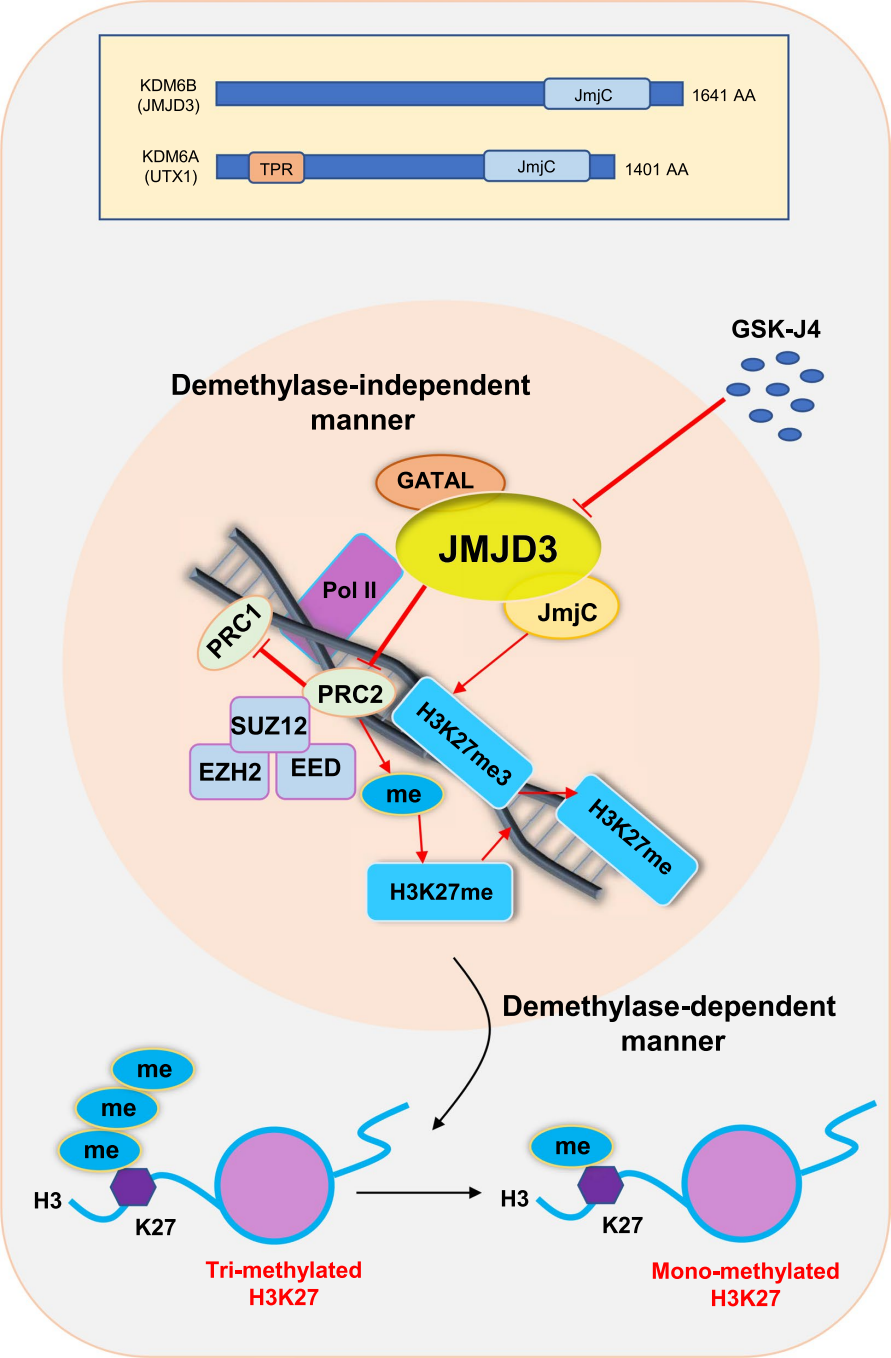
The molecular mechanisms of JMJD3-mediated transcription of target genes. The Jumonji domain-containing protein-3 (JMJD3) belongs to a subfamily of the UTX/UTY JmjC-domain protein. UTX1 (KDM6A) and JMJD3 (KDM6B) are the KDM6 family members. JMJD3 is a histone demethylase that regulates H3K27me3-mediated gene repression and removes H3K27me3. JMJD3 has a JmjC domain (demethylates histones) and a C-terminal segment that is embedded with a GATA-like (GATAL) domain. The KDM6A protein has a catalytic JmjC domain at the C terminus and six tetratricopeptide repeat (TPR) domains at the N terminus. JMJD3 in a demethylase-dependent or independent manner can regulate gene transcription. Polycomb repressive complex 2 (PRC2, including EZH2, SUZ12, and EED) is a transcriptional repressor that interacts with PRC1 and adds methyl groups to histone H3K27. JMJD3, which opposes the enzymatic activity of the PRC2, regulates the expression of specific genes. JMJD3 as a transcription factor can interact with co-activators and regulate the transcription of target genes

There are at least 6 families of histone demethylases. KDM4B (JMJD2B) and KDM6B (JMJD3) are two important histone demethylases [35–37]. Human JMJD3 or KDM6B (lysine-specific demethylase 6B) gene is located at 17p13.1 and encodes a polypeptide that contains 1682 amino acids with an average molecular weight of 176,632 Da [10, 38]. JMJD3 belongs to a subfamily of the UTX/UTY JmjC-domain protein [12]. UTX1 (KDM6A) and JMJD3 are the KDM6 family members that demethylate H3K27me3 [39, 40]. JMJD3 contains a Jumonji C (JmjC) domain (demethylates histones) and a C-terminal segment that is embedded with a GATA-like (GATAL) domain [41, 42]. The KDM6A protein has a catalytic JmjC domain at the C terminus and six tetratricopeptide repeat (TPR) domains at the N terminus [43] (Fig. 1).

JMJD3 catalyzes the transition from a repressive status (H3K27me3) to active status (H3K27me1) [44]. JMJD3 in a demethylase-dependent or independent manner can regulate gene transcription [45]. Polycomb repressive complex 2 (PRC2, composed of the enzyme EZH2, SUZ12, and EED) is a transcriptional repressor that interacts with PRC1 and adds methyl groups to histone H3K27 [46, 47]. JMJD3, which opposes the enzymatic activity of the PRC2, regulates the expression of specific genes [48, 49]. JMJD3 as a transcription factor (independent of demethylase activity) can interact with co-activators and regulate the transcription of target genes [45]. JMJD3 by impacting RNA polymerase II (Pol II) promotes transcriptional elongation and gene expression [50]. The molecular mechanisms of JMJD3-mediated transcription of target genes are shown in Fig. 1.

It has been reported that JMJD3 participates in the regulation of cell cycle arrest, apoptosis, tumorigenesis, immune diseases, and cell differentiation by targeting distinct transcription factors and epigenetic proteins [51–54]. JMJD3 by reducing H3K27me3 enrichment negatively regulates the transcription of Sestrin2 (SESN2) and induces cardiomyocyte apoptosis [55]. JMJD3 also negatively mediates inflammatory response, blood-spinal cord barrier (BSCB) disruption, and apoptosis after spinal cord injury (SCI) [56]. JMJD3 is positively correlated with tumor diameter, differentiation, and microvascular infiltration [10, 57, 58]. Therefore, JMJD3 is involved in cellular plasticity and inhibits abnormal tissue growth by regulating unlimited cell proliferation [59].

## JMJD3 function in embryonic stem cells

JMJD3 plays a critical role in undifferentiated ESCs and ESC-derived cell gene expressions [60]. GSK-J4 by suppressing the enzymatic activity of JMJD3 triggers cell cycle arrest, DNA damage, and cell death in ESCs-derived cells but not in undifferentiated ESCs [60].

**Fig. 2:**
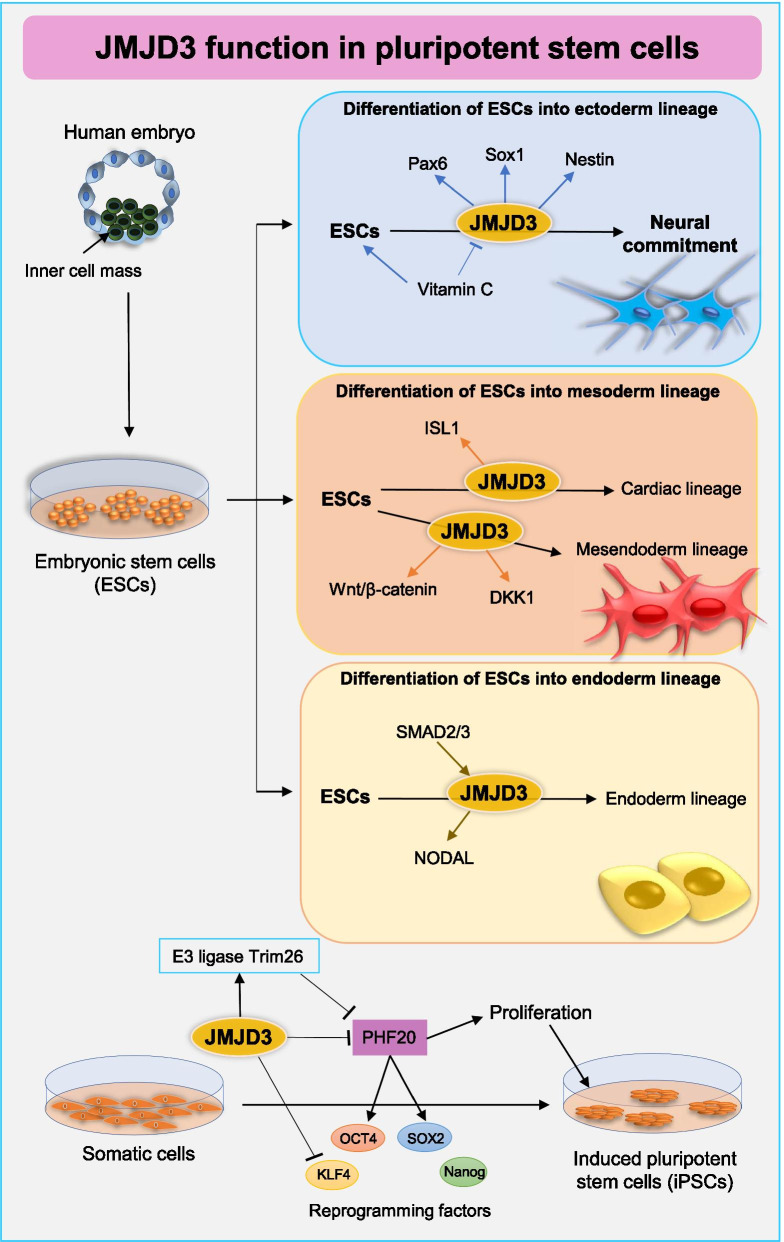
JMJD3 function in pluripotent stem cells. JMJD3 plays a critical role in the differentiation of ESCs and iPSCs. The expression profile of JMJD3 suggests that it may contribute to the regulation of ectoderm, mesoderm, and endoderm differentiation in murine and human ESCs. JMJD3 as an epigenetic barrier is thought to increase during the reprogramming of differentiated cells into iPSCs

The expression profile of JMJD3 suggests that it may contribute to the regulation of ectoderm, mesoderm, and endoderm differentiation in murine and human ESCs [11, 17, 18] (Fig. 2).

- Differentiation of ESCs into ectoderm lineage

During differentiation of ESC to the neuronal lineage, JMJD3 can modulate the expression of key markers of neurogenesis (Pax6, Nestin, and Sox1) and enhance neural commitment [61].

Vitamin C is a critical micronutrient that improves the rate of ESC proliferation and the efficiency of iPSC formation [62]. In mouse ESC differentiation, vitamin C can impact JMJD3 and induce a pluripotent state [63], but during the differentiation of dopamine neurons in the fetal midbrain, vitamin C upregulates JMJD3 and decreases the H3K27m3 of dopamine phenotypic to facilitate dopamine neuron differentiation [64].

- Differentiation of ESCs into mesoderm lineage

JMJD3 also displays an important role in cardiac differentiation [65]. JMJD3 may be involved in the determination of the cardiomyogenic lineage from mouse ESCs and enhanced the expression levels of Jmjd3, Jmjd2a, and Jhdm1d [66]. The transcription factor insulin gene enhancer-binding protein 1 (ISL1) plays a key role in cardiac lineage differentiation [67]. It has been found that ISL1 cooperates with JMJD3 to alter the cardiac epigenome and regulate cardiac differentiation of ESCs [65]. JMJD3 by regulating the Wnt/β-catenin signaling pathway at early and DKK1 (WNT antagonist gene) at a late stage, facilitates differentiation of human ESCs toward mesendoderm and definitive endoderm [32, 68]. Ectopic expression of the JMJD3 has recently been uncovered to facilitate the conversion of hESCs and hiPSCs into skeletal muscle cells and hepatic cells [69].

- Differentiation of ESCs into endoderm lineage

NODAL is the central transcription factor of TGF-β signaling and a target for repression by Polycomb proteins and accumulation of H3K27me3 [70]. SMAD2/3 proteins that transduce signals from TGF-β signaling are capable of recruiting JMJD3 to remove the H3K27me3 repressive mark on the NODAL promoter and facilitate human ESCs differentiation into endoderm [71].

## The role of JMJD3 in reprogramming

JMJD3 as an epigenetic barrier is thought to be increased during the reprogramming of differentiated cells into iPSCs [72] (Fig. 2). It was recently reported that JMJD3 interacts with KLF4 and decreases H3K27me3 in pluripotency genes [34]. A recent study has shown that the Jmjd3-PHF20 axis plays a key role in the reprogramming of somatic cells [33]. PHF20 (plant homeodomain finger protein 20 or glioma-expressed antigen 2) is a critical epigenetic regulator that enhances reprogramming and stemness through activation of Oct4 and Sox2 [73]. JMJD3 by recruiting an E3 ligase Trim26 causes the ubiquitination and degradation of PHF20 [33, 74]. JMJD3 through its H3K27me2/3 demethylase activity enhances the expression of Ink4a/Arf and P21. Thus, a decrease in PHF20 leads to reduce endogenous Oct4 expression, cell proliferation, and the outcome of reprogramming [33]. JMJD3 by removing the H3K27me3 mark from the hepatic transcription factors (HTFs) promoter is participated in the reprogramming of bone marrow progenitor cells (BMPCs) to hepatic cells. In contrast, GSK-J4 can effectively repress the activity of JMJD3 and the loss of the H3K27me3 chromatin mark in BMPCs [75].

## JMJD3 function in multipotent stem cells

- Neural stem cells

It has been reported that JMJD3 interacts with the activated SMAD3 and enhances the differentiation of neural stem cells (NSCs) [76]. JMJD3 is required to interact with neural promoters, regulate neurogenic gene expression, and activate neurogenesis from the adult subventricular zone (SVZ)-derived NSCs [77]. Following differentiation of NSCs to neurons, SMRT (NCoR2, nuclear receptor co-repressor 2) inhibits JMJD3 and maintains the NSCs state [78]. STAT3 as an important component of the LIF signaling pathway is necessary for stem cell self-renewal [79, 80]. STAT3 binds to the JMJD3 promoter, prevents the demethylase activity of JMJD3, and suppresses the activity of differentiation-specific genes [81–83]. It has been shown that inhibition of STAT3 in glioblastoma stem cells (GBM-SC) can promote the levels of histone H3K27 demethylation and the expression of neural-specific genes, such as FGF21, GDF15, and Myt1 [52]. The p53 tumor suppressor has a key role in mouse neurogenesis [84, 85]. In response to differentiation inducers, the recruitment of JMJD3 to p53 responsive elements is increased [86]. During mouse NSCs differentiation, JMJD3 is thought to act as a tumor suppressor and increase the expression of the INK4a/ARF (or CDKN2a) locus, and then stabilize the nuclear distribution of P53 [87].

- Osteogenic stem cells

JMJD3 by removing H3K27me3 plays an important role in the osteogenic commitment of MSCs [88]. JMJD3 appears to induce osteoblast differentiation by stimulating transcription factors Runx2 and Osterix and control the expressions of bone-related genes [44, 89]. Ras-association domain family 5 (RASSF5) or novel Ras effector 1 (NORE1) is a pro-apoptotic protein that regulates a variety of key biological processes [90, 91]. JMJD3 was reported to reduce the expression of RASSF5 and suppress tumor necrosis factor-alpha (TNF-α)-induced osteoblast apoptosis [92].

MicroRNA‐99a by targeting JMJD3 is involved in osteogenic differentiation of bone MSCs [93, 94]. In contrast, MIR146A is a negative regulator of JMJD3 and RUNX2 that reduces MSCs capacity to differentiate into osteoblasts [95]. The PLZF transcription factor was previously shown to play an essential role in the osteogenic fate of human MSCs. At the pre-osteoblast stage of differentiation (osteoblast commitment of progenitor cells), JMJD3 enhances the expression of PLZF and controls osteoblast differentiation in MSCs [96]. Nuclear factor–activated T cells c1 (NFATc1) is a key transcription factor that induces osteoclast differentiation in response to receptor activator of nuclear factor‐κB ligand (RANKL) [66]. JMJD3 has been shown to remove the inhibitory H3K27me3 marker on the Nfatc1 gene and regulate RANKL-mediated osteoclast differentiation [97]. The inhibition of JMJD3 activity by GSK-J4 could be used as a non-invasive treatment for preventing the prefusion of cranial sutures in a patient with excessive osteogenic differentiation of MSCs [98].

- Dental pulp-derived MSCs

JMJD3 by removing H3K27me3 from the promoters of osteogenic genes improves the odontogenic differentiation in dental pulp-derived MSCs [44, 99]. Ethanol (EtOH) can suppress JMJD3 and alter DNA methylation. EtOH-induced DNA methylation influences odontogenic differentiation and reduces mineralization [100]. Insulin‐like growth factor binding protein 5 (IGFBP5) is a multifunctional protein with anti‐inflammatory potential that promotes osteogenic differentiation in dental pulp‐derived MSCs [101, 102]. A recent study showed JMJD3 through the removal of H3K27me3 at the promoter of IGFBP5 mediated periodontal tissue regeneration [101].

- Hematopoietic stem cells

JMJD3 is necessary for the self-renewal properties of hematopoietic stem cells (HSCs) [103].

Unlike KDM6A, which is frequently mutated in hematopoietic disorders, JMJD3 is necessary for HSC self-renewal in response to stress conditions [104, 105]. The adaptor-related protein complex 1 (AP-1) transcription factors such as Fos and JunB are crucial for interleukin (IL)-17-producing T helper (Th17) cell development [106]. JMJD3 was found to modulate the MAPK pathway, suppress the expression of AP-1, and support leukemia initiation and maintenance [107]. Hence, targeted inhibition of JMJD3 led to increaseing HSCs differentiation [74].

These studies suggest that JMJD3 might be a feasible and effective target for cell fate regulation of multipotent stem cells.

## Conclusion

In this review, we summarize the roles of JMJD3 in pluripotency, reprogramming, and differentiation. JMJD3 has been found in several biological processes, including cell proliferation, differentiation, invasion, apoptosis, signaling regulatory pathways. Direct manipulation of epigenomes may be a suitable method for generating desired cell types from pluripotent or multipotent stem cells. Although JMJD3 via epigenetic modifications targets several signaling pathways, off-target effects could lead to minimize the applications of this enzyme in cancer. For example, JMJD3 by promoting cyclin D1 transcription is involved in the development of cancer cells [108]. Also, JMJD3 can impact other histone modifiers and alter chromatin structure, activate the expression of oncogenes, and trigger the development of many types of human diseases [108]. Thus, further studies are required to determine the downstream targets of JMJD3 in pluripotent and multipotent stem cells.

## Data Availability

The datasets used and/or analyzed during the current study are available from the corresponding author on reasonable request.

## Abbreviations

AP-1: Adaptor related protein complex 1
AT: Adipose tissues
BM: Bone marrow
BSCB: Blood-spinal cord barrier
DP: Dental pulp
ESCs: Embryonic stem cells
HSCs: Hematopoietic stem cells
HTFs: Hepatic transcription factors
H3K27me3: Trimethylation of histone H3 on lysine 27
ICM: Inner cell mass
IGFBP5: Insulin‐like growth factor binding protein 5
IPSCs: Induced pluripotent stem cells
JMJD3: Jumonji domain-containing protein-3
KDM6B: Lysine-specific demethylase 6B
LSD1: Lysine-specific demethylase 1
MSCs: Mesenchymal stem cells
NCoR2: Nuclear receptor co-repressor 2
NORE1: Novel Ras effector 1
NFATc1: Nuclear factor–activated T cells c1
PHF20: Plant homeodomain finger protein 20
Pol II: Polymerase II
PRC2: Polycomb repressive complex 2
PSCs: Pluripotent stem cells
RASSF5: Ras-association domain family 5
SCI: Spinal cord injury
SESN2: Sestrin2
SVZ: Subventricular zone
T-ALL: T-cell acute lymphoblastic leukemia
TFs: Transcription factors
TNF-α: Tumor necrosis factor-alpha
UCB: Umbilical cord blood
